# Clinical Outcomes After Viremia Among People Receiving Dolutegravir vs Efavirenz-Based First-line Antiretroviral Therapy in South Africa

**DOI:** 10.1093/ofid/ofad583

**Published:** 2023-11-16

**Authors:** Kwabena Asare, Lara Lewis, Johan van der Molen, Yukteshwar Sookrajh, Thokozani Khubone, Pravikrishnen Moodley, Richard J Lessells, Kogieleum Naidoo, Phelelani Sosibo, Nigel Garrett, Jienchi Dorward

**Affiliations:** Centre for the AIDS Programme of Research in South Africa (CAPRISA), Durban, KwaZulu-Natal, South Africa; Discipline of Public Health Medicine, School of Nursing and Public Health, University of KwaZulu-Natal, Durban, KwaZulu-Natal, South Africa; Centre for the AIDS Programme of Research in South Africa (CAPRISA), Durban, KwaZulu-Natal, South Africa; Centre for the AIDS Programme of Research in South Africa (CAPRISA), Durban, KwaZulu-Natal, South Africa; eThekwini Municipality Health Unit, eThekwini Municipality, Durban, KwaZulu-Natal, South Africa; eThekwini Municipality Health Unit, eThekwini Municipality, Durban, KwaZulu-Natal, South Africa; Department of Virology, University of KwaZulu-Natal and National Health Laboratory Service, Inkosi Albert Luthuli Central Hospital, Durban, KwaZulu-Natal, South Africa; Centre for the AIDS Programme of Research in South Africa (CAPRISA), Durban, KwaZulu-Natal, South Africa; KwaZulu-Natal Research and Innovation Sequencing Platform (KRISP), University of KwaZulu-Natal, Durban, KwaZulu-Natal, South Africa; Centre for the AIDS Programme of Research in South Africa (CAPRISA), Durban, KwaZulu-Natal, South Africa; South African Medical Research Council (SAMRC)-CAPRISA-TB-HIV Pathogenesis and Treatment Research Unit, University of KwaZulu-Natal Nelson R Mandela School of Medicine, Durban, KwaZulu-Natal, South Africa; eThekwini Municipality Health Unit, eThekwini Municipality, Durban, KwaZulu-Natal, South Africa; Centre for the AIDS Programme of Research in South Africa (CAPRISA), Durban, KwaZulu-Natal, South Africa; Discipline of Public Health Medicine, School of Nursing and Public Health, University of KwaZulu-Natal, Durban, KwaZulu-Natal, South Africa; Centre for the AIDS Programme of Research in South Africa (CAPRISA), Durban, KwaZulu-Natal, South Africa; Nuffield Department of Primary Care Health Sciences, University of Oxford, Oxfordshire, UK

**Keywords:** antiretroviral therapy, dolutegravir, retention in care, viremia, viral suppression

## Abstract

**Background:**

We aimed to compare clinical outcomes after viremia between dolutegravir vs efavirenz-based first-line antiretroviral therapy (ART) as evidence is lacking outside clinical trials in resource-limited settings.

**Methods:**

We conducted a retrospective cohort analysis with routine data from 59 South African clinics. We included people with HIV aged ≥15 years receiving first-line tenofovir disoproxil fumarate, lamivudine, dolutegravir (TLD) or tenofovir disoproxil fumarate, emtricitabine, efavirenz (TEE) and with first viremia (≥50 copies/mL) between June and November 2020. We used multivariable modified Poisson regression models to compare retention in care and viral suppression (<50 copies/mL) after 12 months between participants on TLD vs TEE.

**Results:**

At first viremia, among 9657 participants, 6457 (66.9%) were female, and the median age (interquartile range [IQR]) was 37 (31–44) years; 7598 (78.7%) were receiving TEE and 2059 (21.3%) TLD. Retention in care was slightly higher in the TLD group (84.9%) than TEE (80.8%; adjusted risk ratio [aRR], 1.03; 95% CI, 1.00–1.06). Of 6569 participants retained in care with a 12-month viral load, viral suppression was similar between the TLD (78.9%) and TEE (78.8%) groups (aRR, 1.02; 95% CI, 0.98–1.05). However, 3368 participants changed ART during follow-up: the majority from TEE to first-line TLD (89.1%) or second-line (TLD 3.4%, zidovudine/emtricitabine/lopinavir-ritonavir 2.1%). In a sensitivity analysis among the remaining 3980 participants who did not change ART during follow-up and had a 12-month viral load, viral suppression was higher in the TLD (78.9%) than TEE (74.9%) group (aRR, 1.07; 95% CI, 1.03–1.12).

**Conclusions:**

Among people with viremia on first-line ART, dolutegravir was associated with slightly better retention in care and similar or better viral suppression than efavirenz.

In South Africa, about 23% of people with HIV (PWH) experience an episode of viremia during first-line antiretroviral therapy (ART) [1]. First episodes of viremia during ART in PWH are usually due to inconsistent adherence [2, 3] or drug resistance [3–5], and they increase the risk of virologic or treatment failure if adherence is not improved [6, 7]. This often leads to a slower immune reconstitution [8] and a higher incidence of all-cause mortality [9–11].

Dolutegravir is an integrase strand transfer inhibitor (INSTI) being rolled out for ART in South Africa since 2019 [12] and in most low- and middle-income countries (LMICs) [13], replacing the previous drug efavirenz. Compared with efavirenz and other non-nucleoside reverse transcriptase (NNRTI)–based regimens, dolutegravir is more effective and tolerable, with an increased genetic barrier against drug resistance based on clinical trial evidence [14–18]. Accordingly, people receiving first-line dolutegravir-based regimens who present with viremia may be more likely to have inconsistent treatment adherence rather than drug resistance and would be more likely to virally resuppress without the need for changing regimens, unlike people receiving efavirenz [19].

Based on the therapeutic strengths of dolutegravir, the World Health Organization 2021 ART treatment guidelines recommend delaying switching to second-line ART in people with virological failure on first-line dolutegravir-based regimens [20]. This contrasts with recommendations for an early switch to second-line ART among people with virological failure receiving first-line efavirenz and other NNRTI-based regimens [20]. However, there is limited evidence from routine health care settings on clinical outcomes and viral load trajectories after viremia in people receiving first-line dolutegravir-based ART from high–HIV prevalence settings in LMICs.

Therefore, we aimed to assess retention in care and viral load trajectories after viremia in people receiving first-line dolutegravir-based ART compared with those receiving efavirenz.

## METHODS

### Study Design and Setting

We conducted a retrospective cohort study with de-identified, routinely collected data from 59 public, primary health care facilities in eThekwini Municipality, KwaZulu-Natal, South Africa. In these clinics, viral load testing is done at 6 and 12 months after ART initiation and then 12-monthly thereafter [12]. CD4 count is routinely measured at ART initiation and after 12 months and subsequently repeated if clinically indicated (eg, viral load ≥1000 copies/mL).

The 2019 South African HIV treatment guideline [12] recommends that PWH with a viral load ≥50 copies/mL during first-line ART receive enhanced adherence counseling and that a repeat viral load be performed after 2–3 months. People receiving first-line ART with 2 consecutive viral loads ≥1000 copies/mL 2–3 months apart are classified as having virological failure, and switching to second-line ART is recommended if they were receiving an NNRTI-based regimen including efavirenz or nevirapine. However, for those receiving dolutegravir, switching to second-line ART is only recommended after 2 years of ongoing viremia.

### Data Sources and Data Management

The data source for this study was South Africa's TIER.net electronic database, which contains demographics, clinical status, regimen, and clinic visit information of people receiving ART in public sector health care clinics [21]. All data sets were de-identified by the South African National Department of Health's TB/HIV Information Systems (THIS; www.tbhivinfosys.org.za/) before access and use. We performed data cleaning to remove duplicate entries and rationalized ART regimen lines according to clinical guidelines.

### Participants

The study cohort included PWH aged ≥15 years with first viremia (viral load ≥50 copies/mL) between June 1, 2020, and November 30, 2020, while receiving first-line TEE or TLD regimens. The TLD group included participants who initiated TLD or transitioned from an NNRTI-based regimen to TLD before viremia. The ART regimen lines were based on a predefined variable from the TIER.net data set, prevailing guidelines, and clinical considerations. For example, someone who transitioned from TEE to TLD with 2 previous viral loads ≥1000 copies/mL was reclassified as second-line TLD. The ART regimen at viremia was defined as the regimen participants were receiving at the time of viremia, so all participants who transitioned from TEE to TLD on the date of viremia were classified as being on TEE at the time of viremia. We selected the baseline period of viremia to include as many participants as possible on dolutegravir as implementation started in December 2019 and to allow a minimum of 365 days (12 months) plus 90 days of follow-up before the data cutoff of our data set on April 21, 2022. We excluded participants who had been receiving their ART regimen at the time of viremia for <90 days and those not receiving standard first-line regimens of TEE or TLD at the time of viremia.

### Primary Outcomes

Our primary outcomes were retention in care and viral suppression at 12-month follow-up after viremia. Retention in care at 12 months was defined as not being lost to follow-up or recorded in TIER.net as either deceased or “transferred out” to another clinic (as we could not access or link to data at other clinics to establish subsequent retention in care) by 365 days after viremia. Loss to follow-up was defined based on the South African ART program guidelines of being ≥90 days late for a visit [22]. Viral suppression was defined as viral load <50 copies/mL. Considering that viral loads are not always completed on time in routine care, we defined the 12-month window as the closest viral load to 365 days between 181 to 545 days after viremia. Thus, participants with no available viral load within this window were classified as having a missing 12-month viral load and were excluded from the 12-month viral load analysis. We included only the viral loads of participants retained in care.

### Secondary Outcomes

To assess implementation fidelity to the guidelines for managing viremia, we evaluated the secondary outcomes of clinic visit attendance up to 6 months after viremia and 3-month repeat viral load completion and viral suppression (<50 copies/mL). We defined the 3-month viral load window as the closest viral load testing date to 90 days between 28 and 180 days after viremia. Participants with no viral load within this window were classified as having a missing 3-month viral load and were excluded from the 3-month viral load analysis.

### Exposures

The primary exposure was the first-line ART regimen combination (TLD vs TEE) that participants were receiving at the time of viremia. Potential confounding variables or covariates that we included were participant characteristics at viremia, including age, gender, active tuberculosis disease, viral load category, CD4 count category, and time period of viremia.

### Statistical Analyses

We performed all statistical analyses using R 4.2.0 (R Foundation for Statistical Computing, Vienna, Austria) [23]. We used modified Poisson regression models with robust standard errors adjusting for clustering by clinic [24] to determine the risk ratios of retention in care at 12 months and viral suppression at 3 and 12 months after viremia. In all regression models, we compared TLD vs TEE first-line regimens and adjusted for participant characteristics at viremia: age, gender, active tuberculosis disease, time period of viremia, viral load category, and CD4 count category.

We performed 2 separate sensitivity analyses. The first sensitivity analysis involved the 12-month retention-in-care outcome, where we included participants who were transferred out to another clinic as being retained in care. The second sensitivity analysis involved the 12-month viral suppression outcome, where we excluded participants who changed their ART regimen during the 12-month follow-up.

To better understand management and outcomes among people with high-level viremia, we conducted a secondary analysis in participants with viremia ≥1000 copies/mL. In this subgroup, we plotted a Sankey diagram to graphically present viral load trajectories after viremia and switching to second-line ART (only in the TEE group). We only included participants with complete 3- and 12-month viral load results for the Sankey diagram.

## RESULTS

### Cohort Characteristics at the Time of Viremia

**Figure 1.**
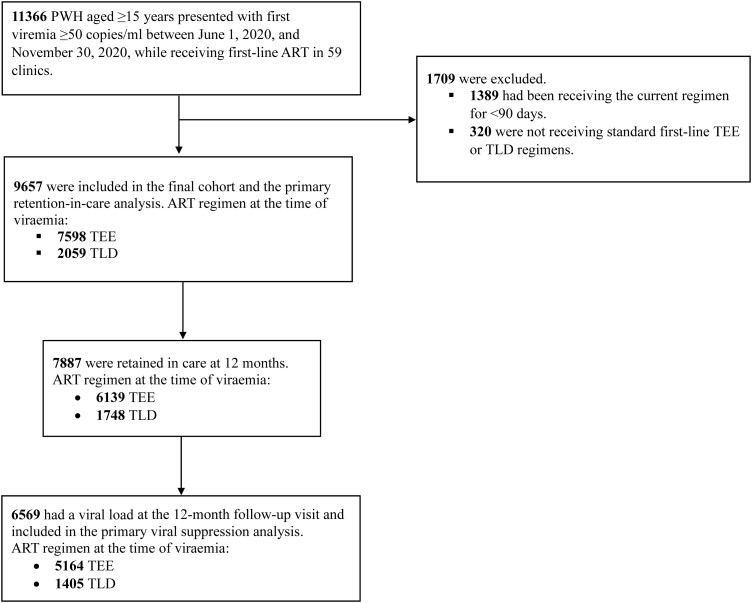
Flow diagram of PWH receiving antiretroviral therapy at 59 clinics in eThekwini Municipality, South Africa. Abbreviations: ART, antiretroviral therapy; PWH, people with HIV; TEE, tenofovir disoproxil fumarate plus emtricitabine plus efavirenz; TLD, tenofovir disoproxil fumarate plus lamivudine plus dolutegravir.

Between June 1, 2020, and November 30, 2020, 11 366 people aged ≥15 years had viremia while receiving first-line ART at the study clinics (Figure 1). At the time of viremia, 1389 had been receiving their current regimen for <90 days, and 320 participants were not receiving standard TEE or TLD regimens and were excluded. Of the remaining 9657 people included in the analyses, 7598 (78.7%) were receiving TEE, and 2059 (21.3%) were receiving TLD regimens at the time of viremia (Table 1). In the TLD group, 584 (28.4%) had been initiated on TLD, and 1475 (71.6%) had transitioned to TLD from an NNRTI-based regimen before viremia. Among the 1475 who had transitioned to TLD from an NNRTI-based regimen before viremia, the most recent (within the past 365 days) viral load (copies/mL) at the time of transition was <1000 in 1029 (69.8%), ≥1000 in only 33 (2.2%), and unavailable in 413 (28.0%). The time on the current regimen (IQR) was lower in the TLD group (0.5 [0.4–0.5] years) than in the TEE group (3.0 [1.1–5.3] years). The median age of the cohort (IQR) was 37 (31–44) years, and 6457 (66.9%) were female, of whom 196 (3.0%) were pregnant. There were more females (n = 5624, 74.0%) in the TEE group and more males (n = 1226, 59.5%) in the TLD group.

**Table 1.** Baseline Characteristics of People With Viremia (≥50 Copies/mL) While Receiving First-line ART

Variable	Overall, n = 9657	ART Regimen at the Time of Viremia	
		TEE, n = 7598	TLD, n = 2059
Age, median (IQR), y	37 (31–44)	36 (30–43)	40 (33–47)
Age			
15–24 y	717 (7.4)	601 (7.9)	116 (5.6)
25–34 y	3234 (33.5)	2727 (35.9)	507 (24.6)
35–44 y	3455 (35.8)	2690 (35.4)	765 (37.2)
45+ y	2251 (23.3)	1580 (20.8)	671 (32.6)
Gender			
Male	3200 (33.1)	1974 (26.0)	1226 (59.5)
Female	6457 (66.9)	5624 (74.0)	833 (40.5)
Known pregnant (females only)	196 (3.0)	188 (3.3)	8 (1.0)
Known active tuberculosis disease	103 (1.1)	69 (0.9)	34 (1.7)
Baseline time period of viremia			
June 2020	730 (7.6)	703 (9.3)	27 (1.3)
July 2020	1116 (11.6)	1018 (13.4)	98 (4.8)
August 2020	2144 (22.2)	1796 (23.6)	348 (16.9)
September 2020	2562 (26.5)	1996 (26.3)	566 (27.5)
October 2020	1704 (17.6)	1222 (16.1)	482 (23.4)
November 2020	1401 (14.5)	863 (11.4)	538 (26.1)
TLD group category			
Started with TLD at ART initiation before viremia	584 (28.4)	…	584 (28.4)
Transitioned from an NNRTI-based regimen to TLD before viremia	1475 (71.6)	…	1475 (71.6)
Most recent viral load (within 365 d) at the time of transition from an NNRTI-based regimen to TLD before viremia			
<1000 copies/mL	1029 (69.8)	…	1029 (69.8)
1000+ copies/mL	33 (2.2)	…	33 (2.2)
Missing	413 (28.0)	…	413 (28.0)
Years since ART initiation, median (IQR)	4.0 (1.1–6.8)	4.0 (1.5–6.9)	3.0 (0.6–6.2)
Years on current ART regimen, median (IQR)	2.0 (0.5–4.9)	3.0 (1.1–5.3)	0.5 (0.4–0.5)
Viral load at viremia			
50–199 copies/mL	4650 (48.2)	3550 (46.7)	1100 (53.4)
200–999 copies/mL	2844 (29.5)	2244 (29.5)	600 (29.1)
1000+ copies/mL	2163 (22.4)	1804 (23.7)	359 (17.4)
Most recent CD4 count			
≤200 cells/μL	1393 (14.4)	1010 (13.3)	383 (18.6)
201–350 cells/μL	1803 (18.7)	1375 (18.1)	428 (20.8)
351–500 cells/μL	1881 (19.5)	1502 (19.8)	379 (18.4)
>500 cells/μL	3194 (33.1)	2638 (34.7)	556 (27.0)
Missing	1386 (14.4)	1073 (14.1)	313 (15.2)
Days since the most recent CD4 count, median (IQR)	708 (196–1462)	718 (204–1463)	507 (186–1453)

Data are No. (%) or median (IQR). Percentages may not add up to 100 because of rounding. All percentages were calculated with the total number in the respective column headers as the denominators unless otherwise stated.

Abbreviations: ART, antiretroviral therapy; IQR, interquartile range, NNRTI, non-nucleoside reverse transcriptase inhibitor; TEE, tenofovir disoproxil fumarate plus emtricitabine plus efavirenz; TLD, tenofovir disoproxil fumarate plus lamivudine plus dolutegravir.

### Clinical Outcomes After Viremia

Twelve months after viremia, 1183 (12.3%) participants were recorded as lost to follow-up, 59 (0.6%) had died, 528 (5.5%) had transferred out to another clinic, and 7887 (81.7%) were retained in care (Table 2). Retention in care at 12 months was 80.8% (n = 6139) in the TEE group and 84.9% (n = 1748) in the TLD group (Table 2). In the multivariable Poisson regression analysis adjusted for age, gender, active tuberculosis disease, time period of viremia, viral load category, and CD4 count category, all at the time of viremia, 12-month retention in care was slightly higher in the TLD group than in the TEE group (adjusted risk ratio [aRR], 1.03; 95% CI, 1.00–1.06; *P* = .047) (Table 3). In the sensitivity analysis (Supplementary Table 1), where we classified people who were transferred out to another clinic as being retained in care, 12-month retention in care was similar between the TLD (89.1%) and TEE (86.6%) groups (aRR, 1.02; 95% CI, 0.99–1.04; *P* = .149).

**Table 2.** Follow-up Outcomes After Viremia (≥50 Copies/mL) in People Receiving First-line ART

Variable	Overall, n = 9657	ART Regimen at Viremia	
		TEE, n = 7598	TLD, n = 2059
At least 1 ART visit within 6 mo	8886 (92.0)	7038 (92.6)	1848 (89.8)
No. of ART visits within 6 mo, median (IQR)	3 (2–3)	3 (2–4)	3 (2–3)
Days to first ART visit within 6 mo, median (IQR)	85 (70–108)	85 (69–107)	86 (77–111)
Repeat 3-mo viral load test done^a^	3741 (42.1)	2961 (42.1)	780 (42.2)
Days to repeat 3-mo viral load, median (IQR)	113 (89–145)	112 (87–141)	120 (96–161)
Repeat 3-month viral load			
<50 copies/mL	2538 (67.8)	1962 (66.3)	576 (73.8)
50–199 copies/mL	445 (11.9)	344 (11.6)	101 (12.9)
200–999 copies/mL	288 (7.7)	241 (8.1)	47 (6.0)
1000+ copies/mL	470 (12.6)	414 (14.0)	56 (7.2)
ART regimen change within 12 mo	3368 (34.9)	3323 (43.7)	45 (2.2)
ART regimen line changed to (of participants who changed regimen)			
1	3074 (91.3)	3040 (91.5)	34 (75.6)
2	294 (8.7)	283 (8.5)	11 (24.4)
Days to ART regimen change within 12 mo (of participants who changed regimen)	189 (84–271)	189 (83–272)	196 (113–253)
ART regimen changed to within 12 mo (of participants who changed regimen)			
TEE (1st-line)	16 (0.5)	0 (0.0)	16 (35.6)
TLD (1st-line)	3001 (89.1)	3001 (90.3)	0 (0.0)
Other (1st-line)	57 (1.7)	39 (1.2)	18 (40.0)^b^
AZT/XTC/LPV/r (2nd-line)	72 (2.1)	66 (2.0)	6 (13.3)
AZT/XTC/DTG (2nd-line)	81 (2.4)	78 (2.3)	3 (6.7)
TLD (2nd-line)	116 (3.4)	116 (3.5)	0 (0.0)
Other (2nd-line)	25 (0.7)	23 (0.7)	2 (4.4)
Follow-up outcome at 12 mo			
Lost to follow-up	1183 (12.3)	967 (12.7)	216 (10.5)
Died	59 (0.6)	50 (0.7)	9 (0.4)
Transferred out to another clinic	528 (5.5)	442 (5.8)	86 (4.2)
Retained in care	7887 (81.7)	6139 (80.8)	1748 (84.9)
12-mo viral load done (of participants in care)	6569 (83.3)	5164 (84.1)	1405 (80.4)
Days to 12-mo viral load, median (IQR)	363 (320–384)	363 (325–384)	363 (301–383)
12-mo viral load			
<50 copies/mL	5175 (78.8)	4067 (78.8)	1108 (78.9)
50–199 copies/mL	570 (8.7)	426 (8.2)	144 (10.2)
200–999 copies/mL	403 (6.1)	316 (6.1)	87 (6.2)
1000+ copies/mL	421 (6.4)	355 (6.9)	66 (4.7)

Data are No. (%) or median (IQR). Percentages may not add up to 100 because of rounding. All percentages were calculated with the total number in the respective column headers as the denominators unless otherwise stated.

Abbreviations: ABC, abacavir; ART, antiretroviral therapy; AZT, zidovudine; DTG, dolutegravir; D4T, stavudine; IQR, interquartile range; LPV/r, lopinavir/ritonavir; NVP, nevirapine; TEE, tenofovir disoproxil fumarate plus emtricitabine plus efavirenz; TLD, tenofovir disoproxil fumarate plus lamivudine plus dolutegravir; XTC, emtricitabine or lamivudine.

^a^Window 1–6 mo.

^b^ABC/XTC/DTG, ABC/XTC/EFV, D4T/XTC/DTG, and TDF/XTC/NVP.

**Table 3.** Univariable and Multivariable Poisson Regression Models of Factors Associated With Retention in Care at 12-Month Follow-up After Viremia (≥50 Copies/mL) in People Receiving First-line ART (n = 9657)

Variable	Level	Retention in Care at 12-Month Follow-up, n/N (%)	Unadjusted RR (95% CI)	*P* Value	Adjusted RR^a^ (95% CI)	*P* Value
First-line regimen at viremia	TEE	6139/7598 (80.8)	1	…	1	…
	TLD	1748/2059 (84.9)	1.04 (1.01–1.07)	.012	1.03 (1.00–1.06)	.047
Age at viremia	15–24 y	485/717 (67.6)	1	…	1	…
	25–34 y	2479/3234 (76.7)	1.13 (1.08–1.18)	<.001	1.11 (1.07–1.16)	<.001
	35–44 y	2939/3455 (85.1)	1.25 (1.19–1.30)	<.001	1.22 (1.17–1.27)	<.001
	45+ y	1984/2251 (88.1)	1.29 (1.22–1.35)	<.001	1.25 (1.19–1.30)	<.001
Gender	Male	2573/3200 (80.4)	1	…	1	…
	Female	5314/6457 (82.3)	1.03 (1.01–1.06)	.007	1.05 (1.02–1.08)	<.001
Known active tuberculosis disease at viremia	No	7821/9554 (81.9)	1	…	1	…
	Yes	66/103 (64.1)	0.78 (0.69–0.89)	<.001	0.84 (0.74–0.97)	.014
Time period of viremia	June to July 2020	1431/1846 (77.5)	1	…	1	…
	August 2020	1799/2144 (83.9)	1.07 (1.03–1.11)	<.001	1.03 (0.99–1.07)	.143
	September 2020	2173/2562 (84.8)	1.09 (1.05–1.12)	<.001	1.04 (1.01–1.07)	.021
	October 2020	1376/1704 (80.8)	1.03 (1.00–1.07)	.051	1.01 (0.98–1.04)	.623
	November 2020	1108/1401 (79.1)	1.01 (0.97–1.06)	.541	0.99 (0.95–1.04)	.770
Recent viral load at viremia	50–199 copies/mL	4087/4650 (87.9)	1	…	1	…
	200–999 copies/mL	2400/2844 (84.4)	0.96 (0.94–0.98)	<.001	0.96 (0.94–0.98)	<.001
	1000+ copies/mL	1400/2163 (64.7)	0.74 (0.71–0.78)	<.001	0.77 (0.73–0.80)	<.001
Recent CD4 count at viremia	≤200 cells/μL	1088/1393 (78.1)	1	…	1	…
	201–350 cells/μL	1466/1803 (81.3)	1.04 (1.01–1.08)	.018	1.02 (0.99–1.05)	.291
	351–500 cells/μL	1555/1881 (82.7)	1.07 (1.04–1.10)	<.001	1.03 (1.00–1.05)	.037
	>500 cells/μL	2685/3194 (84.1)	1.08 (1.05–1.12)	<.001	1.03 (1.00–1.06)	.029
	Missing	1093/1386 (78.9)	1.02 (0.97–1.06)	.461	1.02 (0.98–1.05)	.381

Data are n/N (%) unless otherwise stated.

Abbreviations: ART, antiretroviral therapy; RR, risk ratio; TEE, tenofovir disoproxil fumarate plus emtricitabine plus efavirenz; TLD, tenofovir disoproxil fumarate plus lamivudine plus dolutegravir.

^a^The primary exposure effect (retention in care at 12 mo) is adjusted for all other variables in the table as potential confounders.

Of participants retained in care at 12 months, 6569 (83.3%) had a follow-up viral load done at a median (IQR) of 363 (320–384) days after viremia. By regimen, 5164 (84.1%) in the TEE group and 1405 (80.4%) in the TLD group had a 12-month viral load (Table 2). Of participants with a 12-month viral load, 4067 (78.8%) in the TEE group and 1108 (78.9%) in the TLD group were virally suppressed. The multivariable Poisson regression analysis showed no difference in 12-month viral suppression in the TLD vs TEE group (aRR, 1.02; 95% CI, 0.98–1.05; *P* = .418) (Table 4).

**Table 4.** Univariable and Multivariable Poisson Regression Models of Factors Associated With Viral Suppression at 12-Month Follow-up After Viremia (≥50 Copies/mL) in People Receiving First-line ART who Were Retained in Care at 12-Month Follow-up and Had Viral Load Done (n = 6569)

Variable	Level	Viral Suppression (<50 Copies/mL) at 12 Months, n/N (%)	Unadjusted RR (95% CI)	*P* Value	Adjusted RR^a^ (95% CI)	*P* Value
First-line regimen at viremia	TEE	4067/5164 (78.8)	1	…	1	…
	TLD	1108/1405 (78.9)	1.00 (0.97–1.04)	.915	1.02 (0.98–1.05)	.418
Age at viremia	15–24 y	281/398 (70.6)	1	…	1	…
	25–34 y	1572/2050 (76.7)	1.08 (1.01–1.16)	.022	1.07 (1.00–1.14)	.041
	35–44 y	1979/2480 (79.8)	1.12 (1.06–1.19)	<.001	1.10 (1.04–1.17)	.001
	45+ y	1343/1641 (81.8)	1.15 (1.08–1.23)	<.001	1.13 (1.06–1.20)	<.001
Gender	Male	1631/2086 (78.2)	1	…	1	…
	Female	3544/4483 (79.1)	1.01 (0.98–1.05)	.380	1.01 (0.98–1.04)	.496
Known active tuberculosis disease at viremia	No	5139/6514 (78.9)	1	…	1	…
	Yes	36/55 (65.5)	0.83 (0.70–0.99)	.038	0.89 (0.76–1.05)	.172
Time period of viremia	June to July 2020	968/1229 (78.8)	1	…	1	…
	August 2020	1301/1555 (83.7)	1.07 (1.02–1.11)	.002	1.03 (1.00–1.07)	.077
	September 2020	1476/1845 (80.0)	1.02 (0.97–1.07)	.415	0.99 (0.95–1.03)	.597
	October 2020	822/1109 (74.1)	0.94 (0.90–0.99)	.012	0.92 (0.88–0.97)	<.001
	November 2020	608/831 (73.2)	0.93 (0.87–0.99)	.017	0.91 (0.85–0.97)	.002
Recent viral load at viremia	50–199 copies/mL	2808/3385 (83.0)	1	…	1	…
	200–999 copies/mL	1628/2033 (80.1)	0.97 (0.94–1.00)	.042	0.96 (0.93–0.99)	.023
	1000+ copies/mL	739/1151 (64.2)	0.78 (0.74–0.81)	<.001	0.79 (0.75–0.83)	<.001
Recent CD4 count at viremia	≤200 cells/μL	674/901 (74.8)	1	…	1	…
	201–350 cells/μL	952/1205 (79.0)	1.06 (1.01–1.11)	.023	1.04 (0.99–1.09)	.087
	351–500 cells/μL	1018/1308 (77.8)	1.04 (0.98–1.10)	.170	1.02 (0.96–1.08)	.502
	>500 cells/μL	1838/2246 (81.8)	1.10 (1.04–1.15)	<.001	1.07 (1.02–1.12)	.007
	Missing	693/909 (76.2)	1.02 (0.97–1.07)	.384	1.02 (0.97–1.07)	.419

Data are n/N (%) unless otherwise stated.

^a^The primary exposure effect (viral suppression at 12 mo) is adjusted for all other variables in the table as potential confounders.

Abbreviations: ART, antiretroviral therapy; RR, risk ratio; TEE, tenofovir disoproxil fumarate plus emtricitabine plus efavirenz; TLD, tenofovir disoproxil fumarate plus lamivudine plus dolutegravir.

However, at a median (IQR) of 189 (84–271) days after viremia, 3368 (34.9%) participants changed their ART regimen (Table 2). Of these, 3074 (91.3%) transitioned to another first-line regimen, and 294 (8.7%) switched to second-line regimens. Participants in the TEE group had more regimen changes after viremia (n = 3323, 43.7%) than those in the TLD group (n = 45, 2.2%). In a sensitivity analysis among 3980 participants who did not change their regimen within 12 months after viremia and had a 12-month viral load, 12-month viral suppression was more likely in the TLD group (78.9%) than in the TEE group (74.9%) (aRR, 1.07; 95% CI, 1.03–1.12; *P* = .001) (Table 5).

**Table 5.** Sensitivity Analysis: Univariable and Multivariable Poisson Regression Models of Factors Associated With Viral Suppression at 12-Month Follow-up After Viremia (≥50 Copies/mL) in People Receiving First-line ART who Did Not Change Their ART Regimen Within 12 months, Were Retained in Care at 12-Month Follow-up, and Had Viral Load Done (n = 3980)

Variable	Level	Viral Suppression (<50 Copies/mL) at 12-Month Follow-up, n/N (%)	Unadjusted RR (95% CI)	*P* Value	Adjusted RR^a^ (95% CI)	*P* Value
First-line regimen	TEE	1950/2604 (74.9)	1	…	1	…
	TLD	1086/1376 (78.9)	1.05 (1.01–1.10)	.007	1.07 (1.03–1.12)	.001
Age at viremia	15–24 y	161/246 (65.4)	1	…	1	…
	25–34 y	890/1215 (73.3)	1.11 (1.00–1.23)	.042	1.08 (0.98–1.20)	.122
	35–44 y	1164/1493 (78.0)	1.18 (1.07–1.30)	.001	1.14 (1.03–1.25)	.008
	45+ y	821/1026 (80.0)	1.21 (1.10–1.33)	<.001	1.16 (1.05–1.28)	.004
Gender	Male	1006/1326 (75.9)	1	…	1	…
	Female	2030/2654 (76.5)	1.01 (0.97–1.05)	.527	1.03 (0.99–1.07)	.128
Known active tuberculosis disease at viremia	No	3015/3945 (76.4)	1	…	1	…
	Yes	21/35 (60.0)	0.79 (0.61–1.01)	.056	0.82 (0.66–1.02)	.081
Time period of viremia	June to July 2020	469/640 (73.3)	1	…	1	…
	August 2020	747/917 (81.5)	1.11 (1.05–1.18)	<.001	1.06 (1.00–1.12)	.051
	September 2020	903/1141 (79.1)	1.08 (1.02–1.15)	.010	1.03 (0.97–1.09)	.392
	October 2020	510/712 (71.6)	0.98 (0.92–1.04)	.433	0.93 (0.87–0.98)	.013
	November 2020	407/570 (71.4)	0.97 (0.89–1.06)	.490	0.91 (0.84–0.99)	.031
Recent viral load at viremia	50–199 copies/mL	1715/2107 (81.4)	1	…	1	…
	200–999 copies/mL	940/1222 (76.9)	0.95 (0.91–0.99)	.009	0.94 (0.91–0.98)	.002
	1000+ copies/mL	381/651 (58.5)	0.72 (0.67–0.78)	<.001	0.74 (0.68–0.80)	<.001
Recent CD4 count at viremia	≤200 cells/μL	427/582 (73.4)	1	…	1	…
	201–350 cells/μL	553/720 (76.8)	1.05 (0.98–1.12)	.138	1.04 (0.98–1.10)	.226
	351–500 cells/μL	586/787 (74.5)	1.02 (0.94–1.10)	.637	1.00 (0.93–1.07)	.978
	>500 cells/μL	1077/1344 (80.1)	1.10 (1.04–1.16)	.002	1.08 (1.02–1.13)	.005
	Missing	393/547 (71.8)	0.98 (0.92–1.05)	.604	0.98 (0.92–1.04)	.551

Data are n/N (%) unless otherwise stated.

^a^The primary exposure effect (viral suppression at 12 mo) is adjusted for all other variables in the table as potential confounders.

Abbreviations: ART, antiretroviral therapy; RR, risk ratio; TEE, tenofovir disoproxil fumarate plus emtricitabine plus efavirenz; TLD, tenofovir disoproxil fumarate plus lamivudine plus dolutegravir.

Improving the clinical management of viremia is of critical importance, so we assessed implementation fidelity to viremia management guidelines. In the first 6 months after viremia, 7038 (92.6%) participants in the TEE group and 1848 (89.8%) in the TLD group had at least 1 visit after a median (IQR) of 85 (69–107) and 86 (77–111) days, respectively. Within 6 months, participants in the TEE group had a median (IQR) of 3 (2–4) visits, and participants in the TLD group had a median (IQR) of 3 (2–3) visits (Table 2). Regarding viral load monitoring after viremia, only 2961 (42.1%) participants in the TEE group and 780 (42.2%) in the TLD group had a 3-month repeat viral load done after a median (IQR) of 112 (87–141) and 120 (96–161) days, respectively. Three-month repeat viral load suppression was higher in the TLD group (n = 576, 73.8%) than the TEE group (n = 1962, 66.3%) (aRR, 1.07; 95% CI, 1.02–1.13; *P* = .009) (Supplementary Table 2).

**Figure 2.**
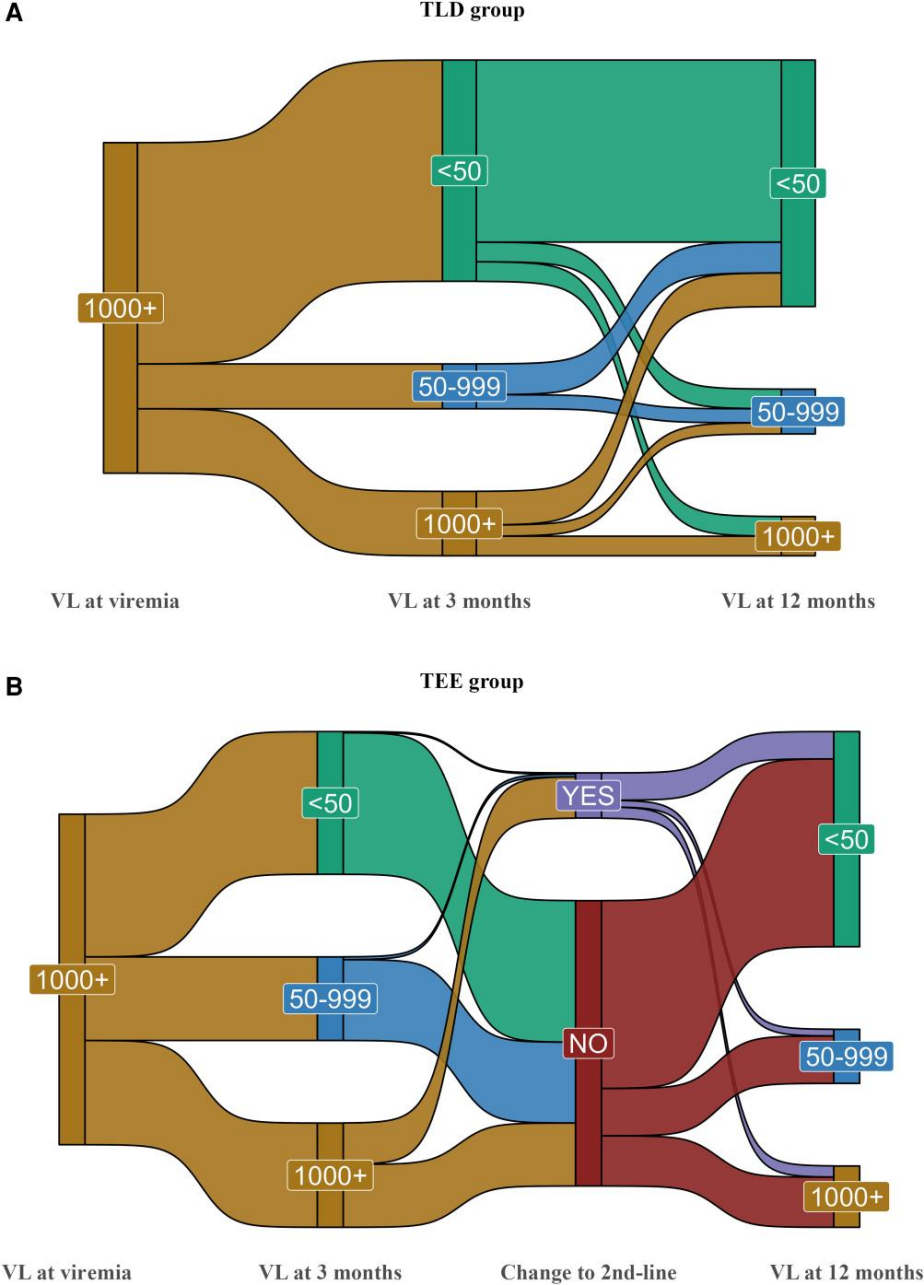
Sankey diagram showing the flow of viremia management outcomes in PWH with high-level viremia (≥1000 copies/mL) while receiving first-line antiretroviral therapy for ≥90 days at the time of viremia. *A*, TLD group. *B*, TEE group. Abbreviations: PWH, people with HIV; TEE, tenofovir disoproxil fumarate plus emtricitabine plus efavirenz; TLD, tenofovir disoproxil fumarate plus lamivudine plus dolutegravir; VL, viral load (copies/mL).

We also assessed follow-up outcomes restricted to participants with high-level viremia (≥1000 copies/mL). A total of 2163 participants presented with high-level viremia, of whom 1804 (83.4%%) were receiving TEE and 359 (16.6%) were receiving TLD (Supplementary Table 3). Of these, 328 (34.7%) participants in the TEE group and 38 (20.0%) in the TLD group had a virological failure (repeat 3-month viral load ≥1000 copies/mL). Of participants with virological failure in the TEE group, 104 (31.7%) were switched to second-line ART within the 12-month follow-up period (from the time of viremia). In the multivariable Poisson regression models, participants who were on TLD at the time of high-level viremia had better outcomes than those on TEE regarding 3-month viral suppression (63.7% vs 42.3%; aRR, 1.39; 95% CI, 1.22–1.58; *P* < .001) (Supplementary Table 4), 12-month retention in care (71.9% vs 63.3%; aRR, 1.11; 95% CI, 1.02–1.22; *P* = .018) (Supplementary Table 5), and 12-month viral suppression (76.6% vs 61.6%; aRR, 1.24; 95% CI, 1.12–1.36; *P* < .001) (Supplementary Table 6). A graphical overview of outcomes after high-level viremia is presented in the Sankey diagram in Figure 2.

In all the Poisson regression analyses, outcomes were generally less likely among participants aged <25 at the time of viremia than older participants.

## DISCUSSION

In this cohort study with large-scale ART programmatic data from 59 public sector health care clinics in South Africa, retention in care after viremia was slightly better among PWH on first-line TLD than TEE. Although the impact of TLD on retention in care is small, every incremental improvement in treatment continuation is important for improving HIV treatment outcomes. Viral suppression after viremia was better among PWH who stayed on first-line TLD than those who stayed on first-line TEE. Retention in care and viral suppression were better with stronger effects on TLD than TEE regimen in the high-level viremia (≥1000 copies/mL) group. Younger people were less likely to be retained in care and achieve viral suppression than older people, and females were more likely to be retained in care.

Limited evidence exists on retention in care and viral suppression after viremia on first-line dolutegravir-based ART in routine LMIC settings, but such data are urgently needed to optimize strategies for managing viremia on dolutegravir. Evidence from clinical trials has shown lower rates of treatment discontinuation and abandonment due to lower rates of adverse events with dolutegravir-based than efavirenz-based ART regimens [17, 18]. Our finding of better retention in care with TLD than TEE after viremia on first-line ART is consistent with the results from these studies [17, 18]. It emphasizes the potential effect of the tolerability of dolutegravir in ensuring consistent treatment adherence and improved clinical outcomes after viremia.

For viral suppression after viremia, we found 1 similar study conducted in South Africa among 385 participants enrolled in the ADVANCE trial who had viremia while receiving first-line dolutegravir-based or efavirenz-based ART regimens [19]. The study used a protocol-defined virologic failure of ≥1000 copies/mL at week 12, ≥200 copies/mL at week 24, or ≥50 copies/mL at week 48 after enrollment. In participants with follow-up viral loads available, viral suppression (<50 copies/mL) within 3 follow-up visits after the first protocol-defined virologic failure was 74.0% (n = 77/104) with tenofovir alafenamide (TAF)/emtricitabine (FTC)/dolutegravir, 85.0% (n = 85/100) with TDF/FTC/dolutegravir, and 40.4% (n = 44/109) with TDF/FTC/efavirenz [19]. This evidence from the ADVANCE trial is consistent with our findings from routine health care settings, but the efavirenz group in the ADVANCE trial had lower viral suppression.

The strengths of our study are that we used large-scale data from routine health care settings, where conditions and outcomes usually differ from clinical trials. We used South African guideline definitions of viremia, retention in care, and viral suppression and adjusted for potential confounders of clinical outcomes after viremia, such as age, gender, active tuberculosis disease, time period of viremia, viral load, and CD4 count. We thus provide robust estimates of retention in care and viral suppression after viremia representative of a real-life, nontrial, and ART program setting.

These findings are essential in resource-limited settings approaching a full rollout to dolutegravir-based regimens for monitoring implementation successes. The findings support the 2019 [12] and current 2023 [25] South African ART treatment guidelines, which recommend the delay of switching to second-line ART after virologic failure among participants receiving first-line dolutegravir-based regimens as enhanced adherence counseling is more likely to lead to resuppression due to dolutegravir's efficacy [14] and increased genetic barrier to drug resistance [26].

Furthermore, our analysis on viremia management revealed health care bottlenecks that can be addressed to improve clinical outcomes after viremia. Although about 90% of participants had a clinic visit within the first 6 months after viremia, less than half had a repeat 3-month viral load. Some of these missing repeat viral loads might have been done but not recorded in TIER.net. However, studies in South Africa [27] and Lesotho [28] that used prospectively collected clinic data have shown similar rates of 47.7% and 40.0% of repeat viral load completion within 6 months after the first elevated viral load ≥1000 copies/mL in participants receiving ART. These gaps lead to missed opportunities for adequate viremia management, such as confirming persistent viremia and switching to second-line ART with potentially negative implications for increased morbidity and mortality [29, 30].

Our analysis also revealed poorer retention in care in males and poorer retention in care and viral suppression outcomes among younger PWH. Younger people remain a high-risk group of the HIV epidemic [31], and these findings indicate that they might also struggle to achieve better treatment outcomes during chronic HIV infection. In South Africa, despite recent improvements in overall HIV treatment outcomes, men record lower HIV health care–seeking behaviors and outcomes than females [32, 33]. Thus, engaging and paying particular attention to male and younger people with viremia on first-line ART might also be necessary as they likely have special needs that must be addressed for improved outcomes.

Our study had some limitations. Although most people currently initiating ART in South Africa are prescribed dolutegravir-based regimens [34], new initiations on dolutegravir and the transition from other regimens to dolutegravir are not random. People initiating or transitioning to dolutegravir may be more likely to be clinically stable than people on different regimens, so we adjusted for the viral load category at the time of viremia in all regression models. However, we cannot rule out confounding by variables not recorded in the data set such as other opportunistic comorbidities and socioeconomic status. The timing of viremia was also different as more participants in the TEE group were viremic during the coronavirus disease 2019 period and might have been at a higher risk of interruptions in care postviremia. However, we adjusted for the time period of viremia in our analyses. This study is also at the early stages of dolutegravir implementation in South Africa, and more extended follow-up data are needed as dolutegravir resistance may increase over time. Our analysis is also limited to 1 province, and a national-level study would be more representative of the South African population. We could not determine whether participants transferred to another clinic were subsequently retained in care or lost to follow-up. This is due to the inability of the current TIER.net database system to effectively link records of participants who change clinics during treatment. This can also be due to some patients being treated as ART-naïve when they have undisclosed ART exposure [35], which in our study could have resulted in participants with previous second-line ART exposure being initiated on first-line regimens. For effective monitoring of HIV and TB outcomes, systems to effectively link participant records when participants change clinics should be developed.

In conclusion, our findings demonstrate that, among people with viremia during first-line ART in routine health care settings, dolutegravir use was associated with slightly better retention in care and better viral suppression among people who did not change ART regimens. Among people with high-level viremia ≥1000 copies/mL, TLD was even more strongly associated with improved retention in care and viral suppression. Improving adherence to guidelines for managing viremia, including enhanced adherence counseling and repeat viral load monitoring, is important for better outcomes with TLD after viremia. Further research to investigate longer-term outcomes, such as switching to second-line ART after 2 years of ongoing viremia on first-line dolutegravir, would be a great addition to the evidence.

## Supplementary Data

Supplementary materials are available at *Open Forum Infectious Diseases* online. Consisting of data provided by the authors to benefit the reader, the posted materials are not copyedited and are the sole responsibility of the authors, so questions or comments should be addressed to the corresponding author.

## Supplementary Material

ofad583_Supplementary_Data

## Notes

***Data availability*.** We cannot publicly share the data used for this study in accordance with the legal and ethical requirements regarding use of routinely collected clinical data in South Africa. Interested parties can obtain access to the data from the eThekwini Municipality Health Unit, Durban, South Africa.

***Patient consent*.** This study was approved by the Biomedical Research Ethics Committee of the University of Kwazulu-Natal (BE646/17), the KwaZulu-Natal Provincial Health Research Ethics Committee (KZ_201807_021), the TB/HIV Information Systems Data Request Committee, and the eThekwini Municipality Health Unit. The ethics approval included a waiver of informed consent for analysis of anonymized, routinely collected data.

***Financial support.*** This work was supported with funding from the Bill and Melinda Gates Foundation (INV-051067). J.D., Academic Clinical Lecturer (CL-2022-13-005), is funded by the UK National Institute for Health and Care Research (NIHR). The views expressed in this publication are those of the authors and not necessarily those of the Bill and Melinda Gates Foundation or the NIHR.
